# Application of a bias-corrected meta-frontier approach and an endogenous switching regression to analyze the technical efficiency of conservation tillage for wheat in South Asia

**DOI:** 10.1007/s11123-018-0525-y

**Published:** 2018-02-05

**Authors:** Sreejith Aravindakshan, Frederick Rossi, T. S. Amjath-Babu, Prakashan Chellattan Veettil, Timothy J. Krupnik

**Affiliations:** 1 Farming Systems Ecology Group, Wageningen University, Wageningen, The Netherlands; 2 International Maize and Wheat Improvement Center (CIMMYT), Dhaka, Bangladesh; 3 University of Florida, Gainesville, USA; 4 Leibniz Centre for Agricultural Landscape Research (ZALF), Muencheberg, Germany; 5 International Rice Research Institute (IRRI), New Delhi, India

**Keywords:** Bangladesh, Bias-corrected meta-frontier, Conservation agriculture, Endogenous switching regression, Meta-technology ratio, Technical efficiency

## Abstract

Conservation tillage (CT) options are among the most rapidly spreading land preparation and crop establishment techniques globally. In South Asia, CT has spread dramatically over the last decade, a result of strong policy support and increasing availability of appropriate machinery. Although many studies have analyzed the yield and profitability of CT systems, the technical efficiency impacts accrued by farmers utilizing CT have received considerably less attention. Employing a DEA framework, we isolated bias-corrected meta-frontier technical efficiencies and meta-technology ratios of three CT options adopted by wheat farmers in Bangladesh, including bed planting (BP), power tiller operated seeding (PTOS), and strip tillage (ST), compared to a control group of farmers practicing traditional tillage (TT). Endogenous switching regression was subsequently employed to overcome potential self-selection bias in the choice of CT, in order to robustly estimate efficiency factors. Among the tillage options studied, PTOS was the most technically efficient, with an average meta-technology ratio of 0.90, followed by BP (0.88), ST (0.83), and TT (0.67). The average predicted meta-frontier technical efficiency for the CT non-adopters under a counterfactual scenario (0.80) was significantly greater (*P* = 0.00) than current TE scores (0.65), indicating the potential for sizeable profitability increases with CT adoption. Conversely, the counterfactual TE of non-adopters was 23% greater than their DEA efficiency, also indicating efficiency gains from CT adoption. Our results provide backing for agricultural development programs in South Asia that aim to increase smallholder farmers’ income through the application of CT as a pathway towards poverty reduction.

**JEL Codes** C06 ● C14 ● Q12 ● C34 ● C51

## 1 Introduction

Over 84% of the globe’s agricultural land is managed by farmers cultivating less than two hectares, the majority of whom focus primarily on the production of staple cereals such as rice, maize, and wheat (FAO 2014). In South Asia alone, these production systems cater to the food needs of over a billion people (cf. Aravindakshan et al. 2015). Production inefficiencies are a particular concern for resource-poor smallholders in South Asia’s rice-wheat rotational systems (Rehman et al. 2014), which occupy over 14 million hectares (Erenstein 2009), as forgone profits resulting from high production costs can determine subsistence above or below the poverty line. Land preparation, tillage, and crop establishment are among the most energy consuming and costly agricultural operations incurred by cereal producers (Gathala et al. 2016; Raghu et al. 2016). Considerable research and development efforts have consequently been undertaken to develop and examine the potential for conservation tillage (CT) technologies and associated crop establishment techniques, which can reduce costs and increase farmers’ production efficiency, while maintaining stable yields (cf. Gathala et al. 2015).

CT is an umbrella term used to describe a portfolio of tillage, crop establishment, and crop residue management systems that aim to conserve soil and water resources, while improving input use efficiency and hence productivity (Abdalla et al. 2013). Cost-savings therefore provide a major driver for CT adoption (Krishna and Veettil 2014). CT examples include zero- and strip-tillage, often with residues retained as mulch, machine-operated shallow-till seeding, and crop establishment on minimally tilled but raised beds reported to increase irrigation use efficiency (Aravindakshan et al. 2015; Gathala et al. 2016), an issue of importance given increasing water pumping costs in Bangladesh (Qureshi et al. 2015). These approaches share similarities with conservation agriculture (CA) that aims to consistently reduce or eliminate tillage (Gathala et al. 2015). But unlike conservation agriculture, farmers practicing CT may not completely eliminate tillage. CT farmers may also remove crop residues for feeding livestock or as cooking fuel (Aravindakshan et al. 2015).

In the last decade, global area under CT has increased by an estimated 60% (Derpsch et al. 2010). The spread of CT has however been most rapid in North and South America, Southern Africa, and in Australia, primarily among larger and wealthier farmers, with less adoption by smallholder farmers (Derpsch et al. 2010). CT is nonetheless a popular technology favored by donors, NGOs, and research organizations in developing nations, and is consequently increasingly promoted as a cost-efficient method of cereal crop production. Several studies have analyzed the determinants of CT adoption in South Asia, though most focus on the western Indo-Gangetic Plains including Uttar Pradesh, Punjab, and Haryana in India, and the Punjab in Pakistan, where farmers tend to be wealthier and have larger field sizes (Krishna et al. 2012; Krishna et al. 2017). Conversely, the eastern Indo-Gangetic Plains (IGP), which is composed of Bangladesh and West Bengal and Bihar in India, tend to be more impoverished, with population densities exceeding 1200 people km^−2^ and considerably smaller field sizes (Erenstein and Thorpe 2011; World Bank 2016). While large production gains and reductions in rural poverty were accrued during the Green Revolution in the eastern IGP, a plateau in factor productivity has been recently been observed (Lin and Huybers 2012). This has retarded the pace of poverty reduction and signaled environmental concerns where inputs are used inefficiently (Erenstein and Thorpe 2011).

Alternative approaches that improve smallholder efficiency, productivity, and profitability may however accelerate poverty reduction process in eastern IGP. CT may therefore have a role to play, while also helping to mitigate environmental externalities and input use inefficiencies prerequisite for sustainable profitability increases (Derpsch et al. 2010; Krishna et al. 2012). The technological efficiency of CT in the poverty-dense eastern IGP has only recently begun to be studied (cf. Keil et al. 2015), although the potential for biases resulting from self-selection in farmers’ technology choices has been insufficiently addressed. This paper therefore responds to this problem, by addressing and overcoming the potential for such confounding artifacts in CT assessments.

Available literature reveals three major short-comings of CT technical efficiency studies. Firstly, previous researchers have presented CT as a single technology, although the term is more appropriate as an umbrella for a number of associated tillage and crop establishment techniques (Abdalla et al. 2013). Studies that have isolated CT into individual tillage options have conversely indicated the risk of self-selection bias (cf. Aravindakshan et al. 2015). Bias resulting from the random selection of villages and households may also be problematic (cf. Krishna and Veettil 2014), as randomization may not ensure an adequate sample of CT adopters and non-adopters for robust analysis. Measured productivity and efficiency variances may therefore be attributed to self-selection rather than unbiased effect (Bravo-Ureta et al. 2012). Finally, few studies have assessed counterfactual effects among CT non-adopters, for example by estimating what efficiency gains may have been accrued had farmers adopted CT, and vice versa for non-adopters.

In this paper, we analyze CT wheat farmers’ technical efficiency (TE) in a representative area of the eastern IGP in northwestern Bangladesh, while addressing the shortcomings of previous analyses. We consider three of the most popular CT technologies adopted by farmers, including bed planting (BP), strip tillage (ST), and reduced tillage machine-aided line sowing using a PTOS or power-tiller operated seeder (see Krupnik et al. 2013 for further description). These are contrasted with farmers’ traditional practices of repetitive tillage, broadcast seeding and crop establishment. The technical efficiency of CT compared to traditional tillage (TT) is analyzed using meta-technology ratios generated via a bootstrapped non-parametric meta-frontier approach that corrects for sampling errors. Bootstrapped truncated regressions are subsequently employed on group-specific efficiency scores to identify the factors affecting TE within tillage options. We subsequently employ endogenous switching regression to correct self-selection biases and to generate counterfactual scenarios. These are matched with meta-frontier technical efficiency scores to reconfirm the impact of farmers’ technology choices.

## 2 Conservation tillage in eastern IGP

Considering the small farm size and poverty in eastern IGP (Erenstein 2009; Krishna et al. 2012), developments in CT technologies have tended to focus on scale appropriate *two-*rather than *four*-wheeled tractors, and attachable land preparation and direct seeding implements (Krupnik et al. 2013). In Bangladesh, two-wheeled tractors were first introduced in the 1980s, while widespread adoption began in the 1990s. Today, the majority of Bangladesh’s land is prepared by two-wheeled tractor (Mottaleb et al. 2016). Various direct seeding and bed planter attachments have been made commercially available for two-wheel tractors in Bangladesh, enabling an increase in CT adoption (Krupnik et al. 2013). Such machinery is normally supplied by machinery service providers through fee for service arrangements. Conversely traditional tillage (TT) techniques in Bangladesh typically include a sequence of three-to-four soil inversion operations with a two-wheel tractor power tiller without direct seeding or bed forming attachments, and with most crop residue removed. Wheat farmers then hand broadcast seeds and incorporate them with another tillage pass, though using similar fee-for-service arrangements.

In this paper, practices included under the rubric of CT include minimal (or reduced) and shallow tillage, single or double pass ridge (or bed) planting, strip tillage, and zero tillage, all using machine-aided direct seeding in rows (Mitchell et al. 2009). Although residue retention is considered a core component of CT sine qua non (Heimlich 1985; Mitchell et al. 2009), many Bangladeshi farmers have not adopted this component. Rather, residues tend to be removed from the field and used as fodder and/or fuel (Aravindakshan et al. 2015). Given these circumstances, we therefore use the term CT referring to the practice of reducing the frequency of tillage passes in conjunction with the usage of mechanical seeding equipment (Table 1).

**Table 1:** Wheat tillage options considered in the study

Tillage options considered in the study^a^	Description
Bed planting (BP)	Beds of approximately 700–740 mm width are established using a two-wheel tractor (2WT) driven bed planter. Wheat is established on beds by direct seeding. Beds are formed with reduced (single or double) tillage, or are permanent requiring only pre-season reshaping
Power tiller operated seeder (PTOS)	The PTOS is a 2WT seed drill attachment that allows shallow tilling and sowing simultaneously. The width of the seeding operation is usually 1200 mm, accommodating six rows of wheat
Strip Tillage (ST)	Tillage is restricted to 40 mm wide strips in 200 mm separated rows by removal of half of the tines from the PTOS. Seed is deposited into these strips while the remainder of the soil surface is untilled. Seeding is completed in a single pass. ST usually requires herbicide application prior to seeding to control weeds
Traditional Tillage (TT)	Traditional tillage with 3–4 passes using a 2WT power tiller, occasionally followed by pre-sowing irrigation, hand broadcast seeding, and then an additional pass to incorporate seed

a For further details, see Krupnik et al. (2013) and Gathala et al. (2016)

## 3 Analytical framework

### 3.1 Measuring tillage adoption impact on farming efficiency

Technical efficiency (TE) improvement can be defined as the ability of an economic unit to produce a given bundle (fixed) of output for a maximum reduction of inputs (Färe et al. 1994). Estimating TE of technology adoption involves the application of either one of the two general approaches: parametric stochastic frontier analysis (SFA) and/or non-parametric data envelopment analysis (DEA). Rahman et al. (2009) and Wollni and Brümmer (2012) recently employed SFA with a selection model proposed by Greene (2010) to analyze the TE of rice and coffee farms in Thailand and Costa Rica, respectively. To account for observed and unobserved variable biases in technology adoption, several recent studies utilize propensity score matching (PSM) with SFA frameworks to correct selection bias (Villano et al. 2015; González-Flores et al. 2014; Bravo-Ureta et al. 2012). Although PSM eliminates a larger proportion of the baseline differences between adopters and non-adopters, its ability to account for unobservable factors such as farmers’ inherent skills and individual capabilities is limited. This can add bias and model dependence. King and Nielsen (2016) recently showed that even when the selection model is balanced and inclusive, PSM can increase imbalance and bias due to approximation of a completely randomized experiment, rather than a more efficient fully blocked randomized experiment.

Out of the two available approaches for TE estimation, we apply an input-oriented DEA model (Banker et al. 1984) to estimate TE gains of wheat farmers using different CT practices. The DEA approach has advantages as there is no requirement to specify a functional form, which is highly restrictive to subsistence level farming context, or to include the prices of the factors of production (Färe et al. 1985; Seiford and Thrall 1990). Using this approach, the frontier is calculated through a piecewise linear envelopment of observed input-output combinations by employing scaling and disposability assumptions (Olesen and Petersen 1995). A farmer qualifies as technically efficient (i.e. it lies on the ‘best practice frontier’ as suggested by Cook et al. 2014), if he or she maintains the current output level (in our case wheat production) using the possible minimum quantity of labor, capital, and technology (nutrients, agrochemicals, seeds, and tillage) inputs. In this study, while farmers manage enterprises consisting of multiple crops—often in rotation with a monsoon *kharif* season rice crop—we focus exclusively on wheat production as wheat tends to be the only crop in the rotation to which CT is widely employed (Erenstein 2009; Aravindakshan et al. 2015; Keil et al. 2015).

Since CT in wheat includes different tillage and crop establishment technology bundles, the estimate of a single frontier for all farmers under different subsets of CT practices is inherently inferior. As such, a meta-frontier (DEA) framework, based on the concept of the meta-production function as an envelope of neoclassical production functions (Hayami and Ruttan 1985), can be used to calculate TE for the global technology and group-specific frontiers for farmers using CT or TT. Technology gaps for farmers following different tillage technologies are then estimated by calculating meta-technology ratio following Battese et al. (2004) and O’Donnell et al. (2008). We therefore employ a bias-corrected DEA meta-frontier estimation, graphically represented and detailed in Figure S1 (see supplementary material). Both group-specific and meta-frontier efficiencies are modeled using wheat yield as the output per farm produced with eight inputs used in wheat production alone. Land, labor, seed, irrigation water, fertilizers, pesticides and fuel are incorporated in physical quantities, tillage machinery use was captured in monetary terms (Table 2).

**Table 2:** Summary statistics of samples showing output and inputs (both per ha and per farm), farm household, management and tillage adoption related variables

Variables	Description unit	TT (*n* = 35) Mean (SD)	BP (*n* = 35) Mean (SD)	PTOS (*n* = 35) Mean (SD)	ST (*n* = 35) Mean (SD)	CT^b^ (*n* = 105) Mean (SD)	Hypothesized direction of impact on efficiency
*Output variable*							
Wheat yield^a^	Quantity of wheat produced by the farm (t farm^−1^)	0.62 (0.35) ***3.68 (0.30)***	0.48 (0.24) ***4.11 (0.69)***	0.96 (1.10) ***4.14 (0.60)***	0.36 (0.23) ***3.79 (0.69)***	0.60 (0.71) ***4.01 (0.68)***	(+)
*Input variables*							
Seed^a^	Quantity of seed (kg farm^−1^)	28.54 (11.55) ***179.39 (31.67)***	13.64 (6.10) ***118.26 (13.53)***	31.49 (35.90) ***134.14 (7.22)***	11.82 (7.07) ***132.67 (43.12)***	18.98** (23.01) ***128.35 (27.13)***	(+)
Chemical fertilisers applied^a^	Quantity of nitrogen, phosphorous and potassium applied (kg farm^−1^)	46.91 (27.90) ***275.81 (41.44)***	24.83 (12.61) ***218.50 (58.43)***	59.68 (78.68) ***246.99 (46.38)***	22.52 (13.05) ***264.58 (78.82)***	35.68 (49.23) ***243.36 (64.91)***	(+/−)
Fuel use^a^	Quantity of diesel fuel consumed by irrigation pump and tillage machine (L farm^−1^)	10.32 (6.22) ***60.06 (13.62)***	4.99 (3.78) ***40.67 (18.67)***	8.10 (8.14) ***35.92 (8.21)***	3.42 (2.21) ***37.41 (9.75)***	5.50*** (5.63) ***38.00 (13.08)***	(+)
Pesticide use^a^	Pesticides applied including insecticides, herbicides etc. (kg farm−1)	0.08 (0.13) ***0.42 (0.48)***	0.09 (0.34) ***0.55 (1.33)***	0.06 (0.14) ***0.20 (0.27)***	0.13 (0.24) ***1.25 (1.74)***	0.09 (0.25) ***0.67 (1.34)***	(+)
Labour^a^	Quantity of labour (both family and hired labour, hours farm^−1^)	133.19 (51.96) ***942.44 (538.83)***	59.54 (21.20) ***549.97 (168.76)***	112.54 (127.79) ***603.7 (489.86)***	48.51 (29.38) ***601.16 (346.23)***	73.53*** (80.97) ***584.93 (357.17)***	(+)
Irrigation^a^	Volume of irrigation water applied (M^3^ farm^−1^)	303.03 (259.43) ***1759.41 (982.79)***	157.00 (89.86) ***1510.75 (807.95)***	335.97 (631.83) ***1286.21 (789.00)***	127.27 (108.97) ***1361.17 (672.38)***	206.75 (381.59) ***1386.23 (757.30)***	(+)
Wheat area^a^	Land under wheat (Hectare)	0.17 (0.10) ***0.17 (0.10)***	0.12 (0.06) ***0.12 (0.06)***	0.23 (0.26) ***0.23 (0.26)***	0.10 (0.06) ***0.10 (0.06)***	0.15 (0.17) ***0.15 (0.17)***	(+/−)
Tillage^a^	Hiring cost of tillage equipment (USD farm^−1^)	9.62 (5.70) ***57.07 (2.80)***	3.59 (1.76) ***30.31 (0.37)***	7.02 (7.87) ***30.47 (0.21)***	2.92 (1.78) ***30.44 (0.38)***	4.51*** (5.06) ***30.41 (0.34)***	
*Farm household variables*							
Cultivable land owned	Arable land owned by the household (Hectares)	0.50 (0.55)	0.60 (0.47)	0.81 (0.58)	0.59 (0.87)	0.67 (0.66)	(+)
Education of the farmer	Highest level of education attained by the household head (Years)	5.29 (2.64)	7.60 (4.10)	8.14 (3.70)	7.06 (3.61)	7.60** (3.80)	(+)
Age of the farmer	Age of the household head (Years)	47.54 (14.55)	44.86 (14.23)	47.46 (10.83)	44.94 (11.59)	45.75 (12.24)	(+)
Training on conservation tillage	Total number of training programmes on CT attended by the household head (Numbers)	0.60 (0.77)	1.80 (1.28)	1.69 (1.11)	2.54 (2.27)	2.01*** (1.66)	(+)
Association with NGOs	Association of household head with various NGO programmes (e.g. Self-help groups). Ordered variable (1 = active member, 2 = passive member, 3 = non-member)	2.69 (0.63)	1.77 (0.84)	1.60 (0.74)	2.23 (0.88)	1.87** (0.86)	(+/−)
Involvement in farming	Ordered (1=full, 2=partial and 3=no involvement)	1.51 (0.56)	1.26 (0.44)	1.57 (0.65)	1.46 (0.56)	1.43 (0.57)	(+)
Remoteness	Distance from the farm to the main road (kms)	0.30 (0.32)	0.17 (0.22)	0.38 (0.63)	0.17 (0.18)	0.24 (0.41)	(−)
Distance to the nearest source of CT extension advice	Distance from the farm to the nearest extension information source for CT technology (kms)	27.11 (16.91)	5.82 (14.50)	12.26 (15.77)	9.68 (15.21)	9.25*** (15.26)	(−)
Farm household size	Number of members in the household (Numbers)	5.26 (2.47)	4.71 (1.36)	5.43 (3.24)	4.66 (2.25)	4.93 (2.41)	(−)
Off-farm income	Share of off-farm income to the household (%)	20.71 (24.53)	21.43 (25.05)	31.14 (25.73)	24.29 (23.64)	25.62 (24.92)	(−)
Draught animals owned	Number of draught animals owned by the household (Numbers)	0.77 (0.94)	0.51 (0.82)	0.11 (0.47)	0.40 (0.74)	0.34* (0.70)	(−)
*Management related variables*							
Advice from input dealer	Whether received advice from input dealer on input application (if received =1, if not Received=0)	1.23 (1.11)	1.34 (1.39)	1.17 (1.34)	1.46 (1.38)	1.32 (1.36)	(+)
Awareness on soil and water conservation	Whether WRC soil and water management recommendations are practiced (Yes=1,otherwise=0)	0.37 (0.49)	0.74 (0.44)	0.54 (0.51)	0.34 (0.48)	0.54 (0.50)	(+)
*Tillage adoption variables*							
Date of sowing	Number of days between the date of wheat sowing and November 15th. Given the value of one, if sown on or before November 15th.^c^	13.46 (9.21)	4.34 (6.68)	5.91 (7.33)	5.49 (5.27)	5.25*** (6.46)	(+)
CT drill scarcity (dummy)	Whether operations were delayed due to CT drill scarcity (if yes=1,otherwise=0)	0.83 (0.38)	0.69 (0.47)	0.54 (0.51)	0.71 (0.46)	0.65* (0.48)	(−)

Values in bold italics shows the per ha data of farm inputs and output in wheat production

*Source*: Farm household survey (2012)

*Notes*:- ‘∗’, ‘∗∗’, and ‘∗∗∗’ = mean differences (t-test) between CT adopters and non-adopters are significant at the 10, 5, and 1% levels, respectively 1 US$ = 81.66 BDT as per the exchange rates during survey (April 2012)

*CT* Conservation Tillage, WRC Wheat Research Centre, Government of Bangladesh

a Measures of input-output variables on per farm basis in the DEA model

b CT refers to pooled sample of BP, PTOS and ST farmers

c Optimum sowing date recommended by WRC in the study area to evade yield loss to terminal heat stress

#### 3.1.1 Group specific frontier efficiency

We assume that farmers (*DMU_j_*, *j* = 1,…, *n*) use a vector of *m* discretionary inputs *X* = (*x*_1_, …, *x_m_*) to produce wheat (*Y*) by adopting any of the *k* tillage technologies. *k* differs with the group we consider while comparing technical efficiencies, that is *k* = {*TT*, *CT*} when we compare the efficiency between traditional and conservation tillage of wheat, and *k* = {*TT*,*PTOS*,*BP*,*ST*} while we compare specific tillage technologies with each of the other separately^1^. Wheat production can be characterized by an input requirement set (Lovell 1993): *L*(*Y*) = {*X*:(*Y*,*X*) is feasible}. Production technology can be defined as:

T={(X,Y):X∈L(Y)} 1

The Farrell (1957) input-oriented measure of technical efficiency of *DMU_j_* is given by:

$$TE_{j}=\min\left\{\delta :\delta X_{j}\epsilon L\left(Y_{j}\right)\right\}$$ 2

This input-oriented technical efficiency model in Eq. 2 depends on the definition of boundary of the observed production of Y as:

IsoqL(Y)={X:XϵL(Y),ϕX∉L(Y),ϕϵ[0,1)} 3

TE is calculated for the farmer *j* in the tillage technology group *k* using piecewise linear programming approach under the following specifications:

$$TE_{kj}=\underset{\delta \lambda ,}{\min}\delta ,\text{subject}\text{to }\sum_{j=1}^{n}\lambda_{kj}y_{kj}\geq y_{k0},\;\sum_{j=1}^{n}\lambda_{kj}x_{mkj}\leq \delta x_{mk0}\forall m$$ 4

with the assumption of either constant return to scale (CRS)—λ*_kj_*≥ 0 or variable return to scale (VRS) — $\sum_{j=1}^{n}\lambda_{kj}=1,\;\lambda_{kj}\geq 0$. The VRS assumption is better accepted for farmers in the smallholder dominated wheat production system of Bangladesh (this assumption is tested later in this paper). For any individual farmer *j* in the group *k*, 0 ≤ δ*_kj_* ≤ 1 and for any technology group *j*, the average TE scores, δ*_k_* lie between 0 ≤ *δ_k_* ≤ 1.

The DEA procedure ignores noise that can arise from sampling or other types of errors, for example one-off events that can impact farmers’ input use decisions and lead to biased *δ_kj_* estimates (Simar 1992). We employed a bootstrapping technique suggested by Simar and Wilson (2007, 2011) to correct biased TE scores (δ*_kj_*), thereby accounting for the non-zero probability mass at one in any given sample. The bias is computed by estimating the pseudo-efficiency estimates $\left({\hat{\delta }}_{kj}^{*(1)},\;\cdots \;,\;\cdots \;,\cdots ,\;{\hat{\delta }}_{kj}^{*(T)}\right)$ by using simulated data set drawn from the original data set, repeated for *T* times (*t* = 1, 2, …., *T*).

$$\text{The estimated bias, }{\hat{b}}_{k}\;=\;\frac{1}{T}\;{\sum_{t=1}^{T}\hat{\delta }}_{kj}^{*(T)}-\;\delta_{kj}\text{ and }$$ 5

the bias-corrected technical efficiency (BC.TE) score is as follows:

$${\bar{\bar{\delta }}}_{kj}\;=\;\delta_{kj}\;-\;{\hat{b}}_{k}.$$ 6

#### 3.1.2 Meta-frontier efficiency and the meta-technology ratio

The tillage-specific efficiency model (*TE_k_*) described above does not allow the direct comparison of TE between individual CT options and TT because these scores are relative to each group’s own frontier (González-Flores et al. 2014). A meta-frontier model is therefore advantageous where several technologies are compared. Similar to the measurement of group frontier efficiency, we specified an input oriented DEA for meta-frontier efficiency estimation (*TE_G_*). However, instead of defining group frontiers as the boundaries of a restricted technology set in each group (e.g., a given tillage option), these meta-frontier efficiency scores are calculated relative to a global or meta-frontier (MF) defined to be the boundary of an unrestricted technology set (i.e. produced by pooling all the farms pertaining to the studied tillage options).

Let *TE_k_*(*x_mk_*, *y_k_*; *δ_k_*) be the input-oriented TE function for the group-frontier representing the group benchmark technology: *T^k^* (*T^k^* = {*T^TT^*, *T^BP^*, *T^PTOS^*, *T^ST^*}) and *TE_G_*(*x_m_*, y; *δ_G_*) be the distance function of the meta-frontier representing global technology, *T^G^*. The gap between *TE_G_* and *TE_k_*is represented by the meta-technology ratio (MTR), which is defined as the ratio of output of the group-specific production frontier relative to the potential output described by the meta-frontier (Battese et al. 2004). That is, the MTR measures the proximity of the tillage specific group frontier (*T^TT^*,*T^BP^*,*T^PTOS^*,*T^ST^*) to the meta-frontier (*T^G^*) with an unrestricted technology set. The MTR between CT and TT is given by:

$$MTR^{k}(.)\;=\;\frac{1\;-\;TE_{G}(.)}{1\;-\;TE_{k}(.)}\;=\;\frac{1\;-\;({\bar{\delta }}_{G}\;-\;{\hat{b}}_{G})}{1\;-\;({\bar{\delta }}_{k}\;-\;{\hat{b}}_{k})}\;=\;\frac{1\;-\;{\bar{\bar{\delta }}}_{G}}{1\;-\;{\bar{\bar{\delta }}}_{k}}$$ 7

Equation 7 captures productivity differences between different tillage technologies. It is indicative of the efficiency improvement potential of wheat farmers in a specific tillage group, that would be possible if they switched to a better tillage technology practiced by other groups of farmers. For example, a relatively high average MTR for a specific tillage group suggests a lower technological gap between farmers in that tillage group in relation to the all available set of tillage technology represented in the meta-frontier. Significant improvements in TE can be realized by switching to technologies that have a higher MTR, wherever feasible. The feasibility of farmers switching to CT is greatly contingent upon the capacities and capital, technological, information and other constraints of individual farm households and on cultural, biophysical, socio-economic and institutional contexts (Ruttan and Hayami 1984). Nevertheless, we hypothesize that TT farmers adopting CT will generally impart a greater gain in efficiency and shrink the technological gap than farmers switching from one CT technology to another CT.

### 3.2 Factors affecting group-frontier efficiency of farmers

Simar and Wilson (2007) noted that DEA efficiency estimates are biased and serially correlated, which invalidates conventional inference in two-stage approaches employing Tobit or ordinary least squares models (Ramalho et al. 2010). However, as more recently evidenced by Solís et al. (2007) and Bravo-Ureta et al. (2012), parameter estimates of TE may be subjected to endogeneity bias due to self-selection. This requires correction for sample selection in parameter estimation models. In case of estimating group-specific efficiency effects, the samples within a particular tillage technology are not systematically different from one another accounting for any selection bias. Bootstrapped truncated regression therefore appears to be pertinent in our case, which we specified as:

$${\bar{\bar{\delta }}}_{kj}\;=\;\alpha \;+\;Z_{j}\phi \;+\;\varepsilon_{j},\;j\;=\;1,\;\cdots ,\;n$$ 8

where ${\bar{\bar{\delta }}}_{kj}$ is the bias-corrected estimate of group-specific efficiency scores (analyzed separately for BP, PTOS, ST and TT), $\varepsilon_{j}\;∼\;N\left(0,\;\sigma_{\varepsilon }^{2}\right)$ with right-truncation at 1–*Z_jφ_ α* is a constant term and *ZJ* is a vector of farmer/farm specific variables. For more details, see Simar and Wilson (2007, 2011).

### 3.3 Factors affecting tillage adoption and meta-frontier efficiency

 Kneip et al. (1998) pointed out that DEA scores converge slowly and are consistent estimators of true efficiency, but biased downwards. In our approach we addressed this bias using bootstrap procedures as explained in Section 3.1.1 and following Simar and Wilson (2007), while employing bootstrapped truncated regression to estimate the factors influencing group level efficiency. Although bootstrapped truncated regression yields unbiased parameter estimates for the determinants of group level efficiency scores, and is therefore preferred to Tobit models, when it comes to estimating the counterfactual effect of technology adoption on meta-frontier efficiency scores, an endogenous switching regression model performs better. Additionally, unlike the traditional OLS and truncated models such as Tobit, these models do not require the conditional distribution of DEA scores (e.g. switching regression and fractional regression) and can yield better estimates (Ramalho et al. 2010). The former approaches may also lead to biased estimates because the decision to adopt CT is voluntary, yet influenced by farmers’ characteristics. For example, farmers who adopt CT may be systematically different from those who do not. Moreover, unobservable characteristics of a given farmer and their farm affects both the CT adoption decision and the resulting efficiency impacts, generating inconsistent estimates of the effect of adoption on household welfare (e.g., if only the most skilled or motivated farmers choose to adopt, the failure to control for skills may result in an upward bias). In addition, some of the factors determining agricultural technology adoption may also influence efficiency, leading to endogeneity problems.

We estimated a standard endogenous switching regression (ESR) model (Maddala and Nelson 1975; Maddala 1983) to deal with problems presented by both sample selection bias and endogeneity (Heckman 1979; Hausman 1978), allowing for interactions between technology adoption and other covariates (Alene and Manyong 2007). This model has two parts: in the first part, endogeneity due to self-selection is addressed using a probit selection model in which farmers are sorted into those who have adopted conservation tillage and those who have not. The second part of the model focusses on the outcome equations on factors influencing efficiency.

Drawing from Maddala (1983) and Lokshin and Sajaia (2004), a probit selection equation for CT adoption is specified as:

$$CT_{j}^{*}\;=\;\gamma S_{j}\;+\;u_{j},\;with\;{\text{A}}_{j}\;=\;\{\begin{array}{cc} 1 & if\;CT_{j}^{*}\;>\;1\;\\ 0 & otherwise \end{array}$$ 9

where $CT_{j}^{*}$ is the unobservable (or latent) variable for CT adoption; *CT_j_* is the observable counterpart (equal to one if the farmer *j* has adopted either BP, PTOS, or ST for wheat during the cropping season studied, and zero otherwise). *S_j_* are non-stochastic vectors of observed farm and non-farm characteristics determining adoption, and *u_j_* are the random disturbances associated with CT adoption. Among *S_j_*, a particular concern is with the dummy variable for CT machine drill scarcity due to its potential for endogeneity bias in selection equation (Eq. 9). Identifying a valid instrument having high correlation with the CT machine scarcity variable, but with low correlation to the dependent variable $CT_{j}^{*}$, is important in two stage estimations. Given this precondition, the distance from the farm to the nearest place at which farmers can receive CT extension advice was selected as the instrument after testing for exclusion restriction and endogeneity.

We subsequently specified the endogenous switching regression model of CT technical efficiency involving two regimes as:

$$Regime\;1\;:\;{\bar{\bar{\delta }}}_{Gj1}\;=\;\beta_{1}S_{1j}\;+\;\tau_{1j},\;if\;CT_{j}\;=\;0\text{ and}$$ 10a

$$Regime\;2\;:\;{\bar{\bar{\delta }}}_{Gj2}\;=\;\beta_{2}S_{2j}\;+\;\tau_{2j},\;if\;CT_{j}\;=\;1,$$ 10b

where ${\bar{\bar{\delta }}}_{Gj1}$ and ${\bar{\bar{\delta }}}_{Gj2}$ are the meta-frontier technical efficiency scores in the outcome equations, and *S*_1_*_j_* and *S*_2_*j* are vectors of exogenous variables assumed to influence technical efficiency. The vectors *β*_1_, *β*_2_, and *γ* are coefficient parameters to be estimated, with error terms *u_j_*, *τ*_1_*_j_* and *τ*_2_*_j_*. The standard switching regression model assumed no covariance between *τ*_1_ and *τ*_2_ with the following covariance matrix:

$$Cov\;(\tau_{1j},\;\tau_{2j},\;u_{j})\;=\;\left[\begin{array}{ccc} \sigma_{\tau_{1}}^{2} & . & \sigma_{\tau_{1}u}\\ . & \sigma_{\tau_{2}}^{2} & \sigma_{\tau_{2\;}u}\\ . & . & \sigma_{u}^{2} \end{array}\right]$$ 11

The covariance between τ_1_ and τ_2_ is not defined since ${\bar{\bar{\delta }}}_{Gj1}$ and ${\bar{\bar{\delta }}}_{Gj2}$ are never observed simultaneously. In the literature, a two-step ESR procedure involving estimation of probit selection model and outcome equations are employed by Perloff et al. (1998). This approach suffers from heteroskedastic errors when inverse mills ratios from probit equations are inserted manually into outcome equations. The full-information maximum likelihood (FIML) yield consistent estimators in which both the probit selection (Eq. 9) and two regime equations (Eqs. 10a and 10b) are estimated simultaneously (Lokshin and Sajaia 2004; Gkypali and Tsekouras 2015). As noted previously, a benefit of switching regression is the ability to consider a counterfactual scenario by using the parameters of one regime (e.g. 10*a*) to predict values for the other regime (e.g., 10*b*), and vice versa. Such hypothetical predictions assume that the coefficients obtained in the switching regression for CT adopters are unbiased estimates of the effect of CT adoption and hence would also apply to non-adopters were they to adopt the CT technology. Conversely, the coefficients obtained for CT non-adopters would apply to CT adopters to simulate *dis*adoption.

## 4 Study area, sample selection, and summary statistics

Data for the present study was collected during 2012 from farm households (*n* = 140) and tillage machinery operators/service providers (*n* = 35) across 15 villages of three districts (Dinajpur, Rajshahi, and Nilphamari) in northwestern Bangladesh. The shares of cultivable area to the total land area in the studied districts were 63% (Rajshahi), 77% (Dinajpur) and 74% (Nilphamari) (BBS 2014). The study area has a sub-tropical climate characterized by monomodal precipitation and wide seasonal variation in rainfall, high temperatures, and high humidity. Wheat is grown during the cool, dry winter (November to April) “*rabi*” season. At the time of survey, wheat was grown on 27,487 ha in Rajshahi, 21,096 ha in Dinajpur and 4461 ha in Nilphamari (BBS 2014). These districts were selected purposively because they represented the main areas where different conservation tillage (BP, PTOS, and ST) methods were being applied in Bangladesh. Neither farmers’ management practices nor soil, climatic and geographical characteristics of these districts are radically different, making regional grouping possible to compare between CT adopters and non-adopters plausible.

Considering their level of CT adoption, five villages were selected from each of the above districts. Tillage technology adoption formed the basis of sample stratification with farmers selected randomly within each technology group. Lists of continuous adopters of the above three CT options and TT farmers supplied by International Maize and Wheat Improvement Center (CIMMYT) and Wheat Research Centre, Bangladesh (WRC) of the Bangladesh Agricultural Research Institute (BARI) were used for sample selection. From each village, three samples belonging to each tillage options were randomly selected from above list for the survey. The final dataset consists of 140 wheat growers (*n* = 35 each for BP, PTOS, and ST), totaling 105 CT adopters, as well as 35 non-adopters (TT). Fifty samples (13 BP, 12 PTOS, 12 ST and 13 TT) were from Rajshahi, forty seven (12 BP, 12 PTOS, 12 ST and 11 TT) were from Dinajpur, and forty three (10 BP, 11 PTOS, 11 ST and 11 TT) were from Nilphamari. Each farmer was interviewed using a structured questionnaire. The survey was implemented from April-June 2012, beginning shortly after the wheat harvest, as this time period is most conducive for minimizing possible recall bias on quantities of inputs used and output obtained. Originally developed in English, the questionnaire was translated into local language (Bangla) to facilitate the interviews.

### 4.1 Summary statistics of the data

We observed significant differences between CT and TT groups in education, training on CT, and association with NGOs (Table 2). CT farmers had higher wheat yields with significantly lower levels of inputs (seed, fertilizers, fuel, irrigation and labor). On average, CT farmers applied 243 kg ha^−1^ of chemical fertilizers and, which is significantly lower (*P* < 0.001) than TT farmers (276 kg ha^−1^). TT farmers also had a significantly higher (*P* = 0.00) seed rate (179 kg ha^−1^) than CT adopters (128 kg ha^−1^). Under traditional tillage, seeds are hand broadcasted, and therefore farmers use a higher seed rate than recommended (125 kg ha^−1^) to get even spread and coverage. Seed drills attached to the two-wheel tractors on the other hand helps in achieving optimum crop line spacing and better ground coverage expending comparatively lesser amount of seeds. The average labor used for TT operations as 942 h ha^−1^, which is approximately 30% higher than CT adopters (585 h ha^−1^).

CT was introduced in the IGP as a cost reducing technology with resource saving benefits, particularly for saving water by increasing soil water holding capacity through improvements in soil organic matter, while arresting soil erosion (Erenstein 2009). The former characteristic of CT could also partially mitigate the need for frequent irrigation. In the case of BP, furrow rather than flood irrigation is widely recognized to save irrigation water (Gathala et al. 2015, 2016). In our dataset, CT wheat farmers were found to utilize approximately 21% less irrigation water than TT. CT farmers were also more aware of soil and water conservation practices compared to the control group. We also found evidence for earlier wheat sowing, averaging 5.25 days before non-adopters. This observation is important because early sowing is recommended to escape from terminal heat stress that can decrease pollen development and stigma deposition, while also shortening wheat crop duration (Krupnik et al. 2015a). Both reduce grain formation and yield in Bangladesh, and can be partially offset by reduced tillage practices that accelerate crop establishment (Krupnik et al. 2015a, Krupnik et al. 2015b). Wheat yields ranged from 3.13 t ha^−1^ to 4.17 t ha^−1^ with traditional tillage, while those for CT ranged from 2.24 t ha^−1^ to 5.65 t ha^−1^. Among the CT adopters, average yield was highest utilizing the PTOS (4.14 t ha^−1^), closely followed by BP (4.11 t ha^−1^). ST farmers achieved on an average 3.80 t ha^−1^, lowest among the three CT options. Other variables (e.g., size of wheat area) showed no statistical differences between group means.

## 5 Results and discussion

### 5.1 TE of CT adoption: DEA meta-frontier framework approach

**Fig. 1:**
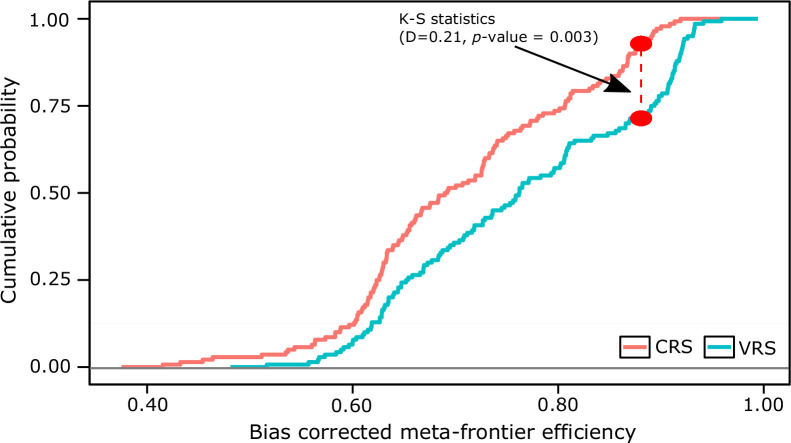
Cumulative probability distribution function, comparing the returns to scale assumption (constant vs. variable return) of the sample data (Kolmogorov–Smirnov test)

Bias-corrected TE scores were estimated for farmers in relation to (i) a specific group’s (BP, PTOS, ST, CT and TT) best practice frontier, and (ii) the meta-frontier of all sampled farms, irrespective of tillage and crop establishment practice. For both frontiers, bootstrapping (10,000 iterations) was conducted^2^. Figure 1 shows the empirical cumulative distribution of meta-frontier DEA models under both VRS and CRS. The *Kolmogorov-Smirnov* test strongly rejected (*P* = 0.003) the CRS model, and hence further discussion of efficiency analyses is based only on the VRS model.

#### 5.1.1 Group-specific *TE*

Results of the bias-corrected group frontiers for each tillage option are reported in Table 3; they provide an indicator of TE with which each of the farmers is operating *within their respective technological group only*. For BP farmers, scores ranged from a low of 0.52 to a maximum of 0.99 (mean = 0.88), while for PTOS farmers the range was 0.64 to 0.99 (mean = 0.85). Among ST farmers, efficiency ranged between 0.68 and 1.00 (mean = 0.90). The wider range of technical efficiency scores of BP farms compared to the relatively narrow TE range for PTOS and ST indicates a high level of operational heterogeneity among BP adopters. The average group-specific mean TE for CT adopters overall is 0.87.

**Table 3:** Meta-technology ratio (MTR) and technical efficiency (TE) for group Frontiers and meta-Frontier

Technical efficiency and technology ratio					
		Mean	Minimum	Maximum	SD
BP	Group TE	0.88	0.52	0.99	0.11
	Meta-frontier TE	0.76***	0.46	0.92	0.12
	MTR	0.88	0.50	1.00	0.13
PTOS	Group TE	0.85	0.64	0.99	0.11
	Meta-frontier TE	0.77***	0.61	0.92	0.11
	MTR	0.90	0.75	1.00	0.07
ST	Group TE	0.90	0.68	1.00	0.10
	Meta-frontier TE	0.74**	0.55	0.95	0.13
	MTR	0.83	0.56	0.98	0.09
TT	Group TE	0.97	0.76	1.00	0.05
	Meta-frontier TE	0.65	0.57	0.74	0.05
	MTR	0.67	0.58	0.80	0.05
CT	Group TE	0.87	0.52	0.99	0.11
	Meta-frontier TE	0.76***	0.46	0.95	0.12
	MTR	0.87	0.50	1.00	0.10

*Note:*- ‘∗∗’, and ‘∗∗∗’ = mean differences between TT and CT options are rejected at the 5, and 1% levels, respectively, by *Wilcoxon–Mann–Whitney* test

BP bed planting, *PTOS* power tiller operated seeding, *ST* strip tillage, *TT* traditional tillage, *CT* conservation tillage

Conversely, the mean group-specific TE for nonadopters (i.e. TT farms) was 0.97, with scores ranging from 0.76 to 1.00. Whereas there were sixteen (46%), eighteen (51%) and fourteen (40%) farms under BP, PTOS and ST operating below 90% efficiency, respectively, there were only three (9%) TT farms operating below this level. Group-specific TE scores can however be highly misleading while comparing across other technology groups as this frontier captures within group variations in technology adoption. For example, TT has already achieved a high level of homogeneity among practitioners as compared to their CT counterparts, translating into a relatively higher group-specific efficiency, part of which may result from farmers’ longer prior experience with TT than CT. While an examination of the average input use to per unit land ratios of TT farmers in Table 2 revealed comparatively small standard deviations (SD) of the mean for total (kg ha^−1^) fertilizer use (SD_TT_ = ±41.44; SD_BP_ = ±58.44; SD_PTOS_ = ±46.38; SD_ST_ = ±78.38) and L ha^−1^ of pesticides (SD_TT_ = ±0.48; SD_BP_ = ±1.34; SD_PTOS_ = ±1.27; SD_ST_ = ±1.74), the standard deviations of the mean for output produced to per unit land ratios for TT were also small (SD_TT_ = ±0.30; SD_BP_ = ±0.69; SD_PTOS_ = ±0.60; SD_ST_ = ±0.69) compared to the high heterogeneity in CT. This indicates a high level of homogeneity in inputs used and output produced by TT followers. TT has been practiced for decades since two-wheeled tractors became the primary tillage option in Bangladesh (Mottaleb et al. 2016); farmers are consequently already highly accustomed in this method of tillage and wheat crop establishment.

More importantly, the group-specific TE of TT farmers are close to the TT group-frontier, which indicates relatively little scope of further improvement within TT. Shifting farmers to alternative TE increasing management practices therefore represents one potential pathway to increase wheat productivity. Considered collectively, the potential for CT farmers to improve is 13%. When compared to the best practices within their own group, however, BP, PTOS, and ST farmers could likely save on average input resources by 12, 15, and 10%, respectively, while maintaining their current production efficiency. The wide range of group-specific TE scores within tillage groups is also indicative of farmers’ deviation and adaptation from recommended CT practices while during the adoption process. Partial adoption of CT has also been reported in the western IGP by Krishna and Veettil (2014). The relative position of TT farmers *viz-a-viz* the meta-frontier is important while exploring more options, but the group-specific frontier does not permit cross-group comparisons. The TE of individual CT groups and the TT group must therefore be compared based on meta-frontier estimates.

#### 5.1.2 Meta-frontier efficiency

**Fig. 2:**
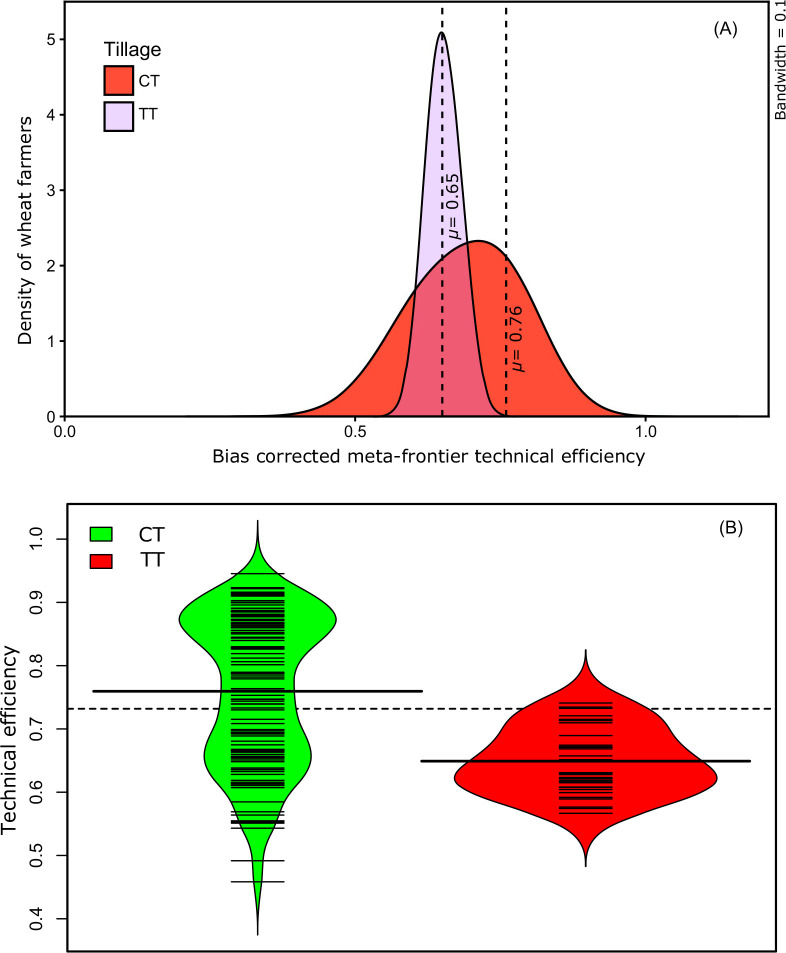
**a** Epanechnikov kernel density estimates of bias corrected meta-frontier TE of CT adopters and non-adopters. ‘*μ*’ denotes the mean values of bias corrected meta-frontier TE. Density curves with orange and purple filled area show bias corrected meta-frontier TE of CT and TT respectively. In each curve, the black dotted vertical line represents the mean value of bias corrected meta-frontier TE. b Technical efficiency of conservation tillage (CT) vs. traditional tillage (TT). Bean shape is a mirror image of the variable’s density plot, aligned vertically. The dotted line represents the threshold technical efficiency (TE) score referring to the mean TE of the entire sample without technology differentiation. Small horizontal lines inside the beans for each tillage option correspond to TE of each farmer. Thin long horizontal lines show the frontier efficiency under each tillage category, while the bold long horizontal lines show the mean TE of farmers in each tillage options

Bias-corrected meta-frontier TE scores of farmers relative to the global meta-frontier are also presented in Table 3. The scores of adopters of any CT technology ranged from as low as 0.46 to a high of 0.95, while that of non-adopters ranged from 0.57 to 0.74. A more informative picture is provided by comparing density curves to illustrate the density distributions of the CT adopters and non-adopters (Fig. 2a). A large number of the CT adopters occupy the meta-frontier technical efficiency range of 0.74 to 0.90, while the density of TT farms is systematically lower than CT farms.

In Fig. 2b, the “heads” of the bean density plots of adopters and non-adopters project in opposite directions. Approximately 97% of the non-adopters fall below the threshold TE score of 0.74, while approximately 42% of CT adopters are above the threshold.^3^ This indicates the superiority of CT from a TE standpoint. The association between CT adoption and TE in wheat cultivation is further examined using the *adapted*-Li test (cf. Simar and Zelenyuk 2006), which compares the equality of distributions of adopters and non-adopters across TE ranges. This test confirms significant differences (*P* ≤ 0.001) between the TE distributions of CT adopters and non-adopters after 10,000 bootstrap iterations (Table 4).^4^

**Table 4:** Testing equality of technical efficiency distributions and density estimates

A. Test for equality of efficiency distributions^a^			B. Epanechnikov Kernel density^b^		
Null hypothesis	Li Test (*T_n_*)	*p*-value	Tillage	Max. density	*Peak*-value
*Distribution* (BP_scores_) = *Distribution* (TT_scores_)	7.91	<2.22e-16 ***	BP	2.27	0.80
*Distribution* (PTOS_scores_) = *Distribution* (TT_scores_)	3.92	2e-03***	PTOS	2.31	0.76
*Distribution* (ST_scores_)= *Distribution* (TT_scores_)	4.87	2e-04***	ST	1.96	0.75
*Distribution* (CT_scores_) = *Distribution* (TT_scores_)	14.51	<2.22e-16***	TT	5.23	0.65

’***’ = Null of equality of distributions is rejected at the 0.1% level, “*T_n_*” refers to test statistic

BP Bed planting, *PTOS* power tiller operated seeding, *ST* strip tillage, *TT* traditional tillage, *CT* conservation tillage

a Li test (Li et al. 2009) and bootstrap P-value from 10,000 iterations. Estimation in R using “np” package

b Kernel density values of bias corrected meta-frontier technical efficiency scores

**Fig. 3:**
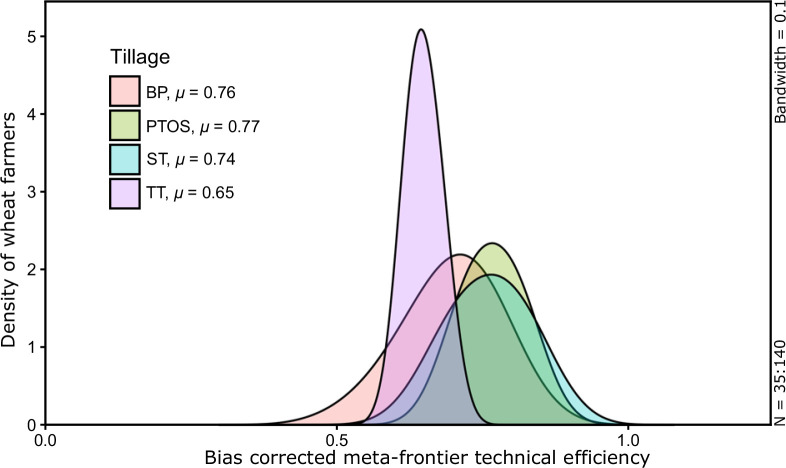
Epanechnikov kernel density estimates of bias corrected meta-frontier TE of tillage options. ‘*μ*’ denotes the mean values of bias corrected meta-frontier TE. Density curves with pink, green, blue and purple filled area show bias corrected meta-frontier TE of BP, PTOS, ST and TT respectively (color figure online)

Figure 3 displays a kernel density plot of the tillage practices utilized by surveyed farmers based on Epanechnikov kernel density estimates from Table 4. As no distributional assumptions were made on the DEA meta-frontier TE scores across the tillage options in this study, this plot type is advantageous for understanding the efficiency gains from TE under each technology. The height of the curves indicates the probability of a farmer achieving a certain efficiency level; the more peaked and narrow distributions indicate limited variance. TT exhibits a peak density of approximately 5.23, covering a bias corrected meta-frontier TE range of 0.57 to 0.74. The maximum density value of the underlying meta-frontier efficiency among the CT options is achieved by PTOS adopters (2.31), closely followed by BP (2.27) and ST (1.96). BP adopters have the broadest distribution ranging from 0.46 to 0.92, indicating higher variance within its practices, though all CT options are relatively similar. Though density of TT is narrow (representing a more homogenous application of tillage and crop management practices), peaking occurs at a lower efficiency level (0.65), whereas the distribution of density of CT options are wider with more flatness (indicating heterogenous adoption). They are however skewed towards a higher efficiency level, peaking at efficiency levels 0.80, 0.76 and 0.75 for BP, PTOS and ST, respectively.

**Fig. 4:**
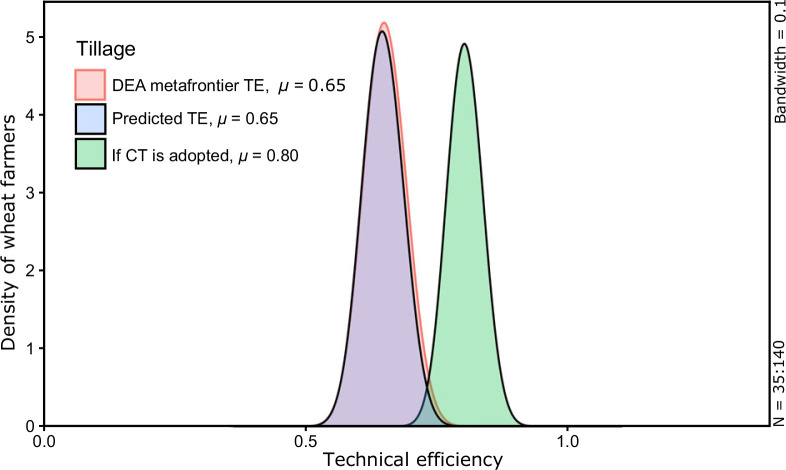
Counterfactual effect of farmers using traditional tillage (TT). The density curves with red outer line and pink shaded area shows estimated bias corrected meta-frontier TE from DEA, while the curve with black outer line and blue shaded area shows the predicted TE from endogenous switching regression. The curve with black outer line and green shaded area presents the TE of TT farmers under a hypothetical scenario if they would have adopted the CT practice (color figure online)

In sum, it is apparent from the mean of bias-corrected meta-frontier TE scores of individual CT technologies that BP and PTOS are more efficient tillage technologies, with average efficiency scores of 0.76 and 0.77, respectively (Table 3), with the Epanechnikov kernel density curves (Figure; the graphical data of Fig. 4) reinforcing this observation. These two groups are closely followed by ST adopters, with an average meta-frontier efficiency of 0.74, whereas TT lags well behind at 0.65.

**Fig. 5:**
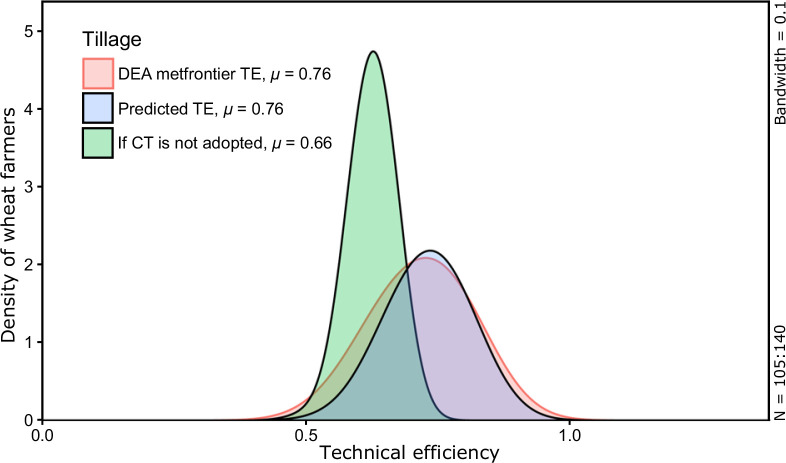
Counterfactual effect of farmers using traditional tillage (CT). The density curves with red outer line and pink shaded area shows estimated bias corrected meta-frontier TE from DEA, while the curve with black outer line and blue shaded area shows the predicted TE from endogenous switching regression. The curve with black outer line and green shaded area presents the TE of CT farmers under a hypothetical scenario if they would have *dis*adopted the CT practice (color figure online)

#### 5.1.3 Measuring the technological gap

On average, CT adopters exhibit a meta-technology ratio (MTR) of 0.87, which is 30% greater than CT non-adopters (Table 3). A higher average MTR for CT relative to CT non-adopters indicates that the former requires a lower level of inputs relative to the latter to achieve same level of output, *ceteris paribus*. If TT farmers were to switch tillage type to either BP, PTOS, or ST, then our data indicate that they could achieve an increase in MTR of 31, 34, and 23%, respectively (Table 5). Concomitantly, the respective increase in TE would be 18, 17and14%, provided that farmers have sufficient knowledge of the alternative options to maintain efficient management. It can be expected that switching from one CT tillage practice to another is less likely to provide sufficient efficiency gains to justify the switch. Regardless of specific tillage and crop establishment type, switching from TT to one of the CT options is however only logically feasible where CT machinery and service provision is available.

**Table 5:** Economic and Technical improvement with CT adoption

	Estimated change resulting from new tillage and crop establishment methods (%)		
	Mean TE change	Change in mean MTR	Change in mean gross margin
From TT to BP	16.92	31.34	190
From TT to PTOS	18.46	34.33	212
From TT to ST	13.85	23.88	125
From TT to CT	16.92	29.85	168

*BP* bed planting, *PTOS* power tiller operated seeding, *ST* strip tillage, *TT* traditional tillage, *CT* conservation tillage

### 5.2 Factors affecting the group-specific TE of individual tillage options

The parameters for the group-specific TE estimated are reported in Table 6. The extent of cultivable land owned had no significant effect on group-specific TE for any tillage and crop establishment option, except for BP (*P* ≤ 0.01). TE of the BP farmers having more than one hectare of cultivable land would be 13.5 percentage points higher than the average BP farmer. With BP, irrigation is channeled through furrows between beds (Qureshi et al. 2015; Gathala et al. 2016). The significant and positive effect on BP may be due to farm size, as farmers may benefit more from the even distribution of irrigation water and ease of intercultural operations offered by BP configurations, than farmers who can provide more careful management to smaller plots. The effect of education is positive on the group-specific TE of CT options (BP, PTOS and ST). This is as expected, though education was significant for CT only (*P* ≤ 0.01). Efficient use of CT practices appears to be pronounced for educated farmers. This is reflected in an observed positive TE effect. Farmers’ age conversely negatively affected the group specific TE of all CT options, though significant only in case of ST (*P* ≤ 0.01). Perhaps unsurprisingly, older TT farmers were more efficient as demonstrated by a positive and significant coefficient (*P* ≤ 0.01). This may be due to longer experience with traditional wheat cultivation practices gained over time. While training on conservation tillage received by BP and ST farmers contributed significantly and positively to farmers’ group specific TE, such training was not significant for PTOS or TT practitioners. Preparation of beds in BP as well as strips and furrows in ST require considerably more skill than planting with the PTOS or broadcasting seeds under TT (Krupnik et al. 2013). Farmers in our dataset who received training reported acquiring skills for forming beds and strip furrows, helping to explain the above positive and significant association.

**Table 6:** Regression results

	Endogenous switching regression (dependent variable (selection eq.): binary treatment variable (0,1) (dependent variable (outcome equations): meta-frontier TE)				Bootstrapped truncated regression (dependent variable: group-frontier TE)			
	Probit selection eq., (*n* = 140)		Outcome eq. 1, TT (*n* = 35)	Outcome eq. 2, CT (*n* = 105)	BP (*n* = 35)	PTOS (*n* = 35)	ST (*n* = 35)	TT (*n* = 35)
	Estimates	Marginal effects						
Model intercept	3.893* (1.648)	–	0.689*** (0.041)	0.821*** (0.041)	1.308** (0.990, 1.630)	0.795*** (0.566, 1.013)	0.208 (−0.329, 0.583)	0.689** (0.525, 0.825)
*Farm household variables*								
Cultivable land owned	0.095 (0.390)	0.016 (0.032)	−0.012 (0.009)	−0.023 (0.014)	0.135** (0.051, 0.236)	−0.040 (−0.100, 0.018)	−0.103 (−0.192, −0.023)	0.004 (−0.024, 0.033)
Education of the farmer	−0.038 (0.063)	−0.002 (0.005)	0.006* (0.003)	0.006** (0.002)	0.043** (0.058, 0.031)	0.020** (0.028, 0.011)	0.022** (0.029, 0.015)	0.010 (−0.001, 0.022)
Age of the farmer	−0.032* (0.018)	−0.002 (0.002)	0.000 (0.000)	−0.001* (0.001)	−0.003 (−0.006, 0.000)	−0.005 (0.002, 0.008)	−0.015* (0.009, 0.023)	0.004** (0.003, 0.005)
Training on conservation tillage	0.726** (0.248)	0.053* (0.030)	0.002 (0.008)	0.024*** (0.005)	0.037* (0.009, 0.068)	−0.004 (−0.041, 0.029)	0.040** (−0.069, −0.017)	0.012 (−0.003, 0.027)
Association with NGOs	−1.754*** (0.486)	−0.138* (0.084)	−0.020 (0.015)	−0.028* (0.015)	−0.151* (−0.217, −0.095)	−0.120** (0.078, 0.164)	−0.070 (0.016, 0.133)	−0.067* (−0.094, −0.043)
Involvement in farming	0.328 (1.381)	0.016 (0.123)	−0.006 (0.023)	0.024 (0.029)	0.082 (−0.040, 0.223)	0.124** (0.057, 0.195)	0.163 (−0.318, 0.031)	0.090*** (0.057, 0.128)
Remoteness	0.179 (0.617)	0.002 (0.046)	−0.067*** (0.019)	−0.018 (0.020)	−0.483* (0.291, 0.695)	−0.082* (0.038, 0.108)	−0.290 (−0.667, 0.039)	−0.223** (0.167, 0.289)
Distance of farm from source of CT extension advice	−0.042*** (0.012)	−0.003* (0.002)	0.000 (0.000)	0.001 (0.001)	0.001 (−0.002, 0.003)	−0.002** (0.005, 0.004)	−0.011** (0.007, 0.017)	−0.002 (0.001, 0.003)
Farm household size	−0.063 (0.099)	−0.003 (0.008)	0.000 (0.002)	0.001 (0.004)	0.005 (−0.023, 0.034)	−0.002 (−0.011, 0.007)	0.019 (−0.006, 0.047)	−0.012* (−0.016, −0.007)
Off-farm income	0.003 (0.009)	0.000 (0.001)	0.000 (0.000)	0.000 (0.000)	−0.001 (−0.004, 0.001)	−0.005 (−0.006, −0.003)	−0.009* (0.006, 0.014)	−0.001 (0.000, −0.011)
Draught animals owned	−0.452* (0.256)	−0.032* (0.028)	−0.008 (0.006)	−0.019* (0.010)	0.023	−0.124	−0.100	−0.032
*Adoption related variables*								
Delayed sowing	−0.096** (0.031)	−0.008* (0.005)	−0.002* (0.001)	−0.006*** (0.001)	−0.015* (−0.006, −0.024)	−0.009* (0.005, 0.014)	0.008 (−0.002, 0.018)	−0.004 (0.001, −0.007)
CT drill scarcity	0.306 (0.617)	0.005 (0.041)	−0.022 (0.017)	−0.055*** (0.015)	0.018 (−0.056, 0.093)	0.051 (−0.019, 0.118)	−0.210 (0.085, 0.371)	−0.050 (−0.111, 0.012)
*Management related variable*								
Advice from input dealer	0.152 (0.162)	0.011 (0.014)	–	–	0.027 (−0.002, 0.058)	−0.032 (0.009, 0.058)	−0.041 (−0.095, 0.010)	−0.046*** (0.035, 0.059)
Awareness on soil and water conservation	−0.309 (0.481)	−0.022 (0.039)	–	–	0.080 (−0.052, 0.216)	−0.072 (−0.162, 0.016)	−0.413 (−0.715, −0.178)	−0.083 (−0.141, −0.032)
*sigma1*	0.025*** (0.003)							
*Sigma2*	0.070*** (0.005)							
*rho1*	0.229 (0.543)							
*rho2*	−0.386 (0.440)							

*Notes*: ∗, ∗∗, and ∗∗∗ indicate statistical significance at the 10, 5, and 1% levels, respectively. Standard errors are shown in parentheses

Dependent variable for probit selection equation: binary treatment variable (0=non-adoption, 1=adoption)

In case of truncated regression estimates, the first entry in each cell is the coefficient. Values in parentheses are the 95 % confidence band (lower and upper) derived from 10,000 bootstrap replications

*BP* bed planting, *PTOS* power tiller operated seeding, *ST* strip tillage, *TT* traditional tillage, *CT* conservation tillage

Memberships in groups formed by NGOs negatively affected group frontiers, irrespective of the tillage and crop establishment option considered, though not significant for ST adopters. NGOs in the study area often promote off-farm and diversified livelihood options including small business entrepreneurship. Loans for farming activities tend to focus on non-cereal cash crops (e.g., vegetables and oilseeds). Farmers associated with NGOs are therefore likely concentrate more on these crops to repay loans, which may influence the TE of wheat production negatively. Conversely, a positive impact on efficiency is expected with farmers’ increasing involvement in agriculture. This variable was positive across all tillage options with significant coefficients for PTOS (*P* ≤ 0.01) and TT (*P* ≤ 0.001).

Fifty five percent of observed farmers sowed wheat after the second week of November. Our data indicate that these TT farmers sowed wheat on average 13.46 (±9.21 SD) days after November 15th, while across CT farmers, this delay was only 5.25 (±6.46 SD) days, resulting in significant sowing date differences (*P* ≤ 0.001) observed for this ensemble group. Krupnik et al. (2015a, 2015b) discuss the physiological basis by which delayed sowing impairs yields in Bangladesh. Krishna and Veettil (2014) also reported a drastic reduction in TE due to late wheat sowing in the western IGP, though the reasons for differences in significance between tillage options require further research. Remotely located farmers were found to be more inefficient, and as the distance of their farms to sources of CT extension advice increases with remoteness. Inefficiency therefore increased with remote PTOS and ST farmers. On the other hand, while off-farm income was found to significantly (*P* ≤ 0.05) decrease the efficiency of ST farmers, larger household size significantly (*P* ≤ 0.05) reduced efficiency of TT. Remaining variables such as ownership of draught animals, seed drill scarcity, and farmers’ awareness of soil and water conservation showed no statistically significant effect on the group-specific TE of any of the tillage options in our data.

### 5.3 Sources of TE and CT adoption: endogenous switching regression estimates

To understand why farmers adopt conservation tillage, and what factors determine their TE, we employed endogenous switching regression (ESR). Among the variables considered in the selection equation of ESR, the presence of potential endogeneity of the variable “CT drill scarcity” on CT adoption was explored by an instrumental variable viz. *distance to the nearest source of CT extension advice*. The latter was found to be strongly correlated with CT drill scarcity (*r* = 0.82), but weakly correlated to adoption (*r* = −0.17). A two-stage IV estimation (*F* > 10.0) followed by a Hausman test rejected presence of any endogeneity bias (*P* = 0.39), suggesting the validity of the selection model used. The results of the probit selection equation and the two outcome equations of the switching regression analysis are reported in Table 6. The former examines the determinants of conservation tillage adoption, while the determinants of technical efficiency for the TT (non-adopters) and CT (adopters) are analyzed by the outcome Equations 1 (Eq. 10a) and 2 (Eq. 10b), respectively.

While education had no effect on the tillage adoption behavior, it had a positive and statistically significant effect on the TE of wheat farmers surveyed, for both CT and TT, at the 1 and 5% level respectively. Compared to a non-literate farmer to a farmer with university education, the TE of tillage adoption differs by 9%. Wadud (2003) reported that literate and more educated farmers are able to better utilize farmer social information and communication networks. This suggests that farmers’ ability to use new technologies and choose optimal input combinations is likely to improve with education. It also indicates the need for developing effective interventions that can improve farmers’ capability to effectively adapt and utilize novel technologies, since the education level of farmers cannot be easily improved over short time periods. Older farmers tended not to favor CT adoption (*P* ≤ 0.05). Those who did adopt CT were also inefficient compared to younger farmers (*P* ≤ 0.05).

Like educational level, agricultural training had a positive and significant effect (*P* ≤ 0.01) on CT adoption. Inference from the marginal effects was also significant (*P* ≤ 0.05); this shows that each time a farmer attends training, the probability of CT adoption increased by 5.3%. Among CT adopters, training had a positive and significant impact (*P* ≤ 0.001) on TE, with a reported increase of 2.4% efficiency per training undergone. Note that such high contribution of training is primarily because of the existing heterogeneous and partial adoption of CT technologies prevalent in the region. Increased proximity to a source of extension information on CT favored CT adoption. These variables show a clear evidence of the impact on adoption of ongoing extension efforts in the study area. The probability of a household located nearby a source of CT extension advice adopting CT was conversely 3% higher than a household located 10 km away from the CT extension source. In contrast, farmers’ engagements with NGOs were found to significantly (*P* ≤ 0.001) and negatively affect CT adoption. A change from non-member to an active member of an NGO reduced the probability of CT adoption by 28%. One potential reason for this observation may be NGO disbursement of microfinance loans linked to specific programs unrelated to agriculture. Conversely, if agricultural loans are available, they are often of limited in size and scope, with an emphasis on cash crop production and at levels too small to enable CT machinery purchase.

Although a positive relationship with TE was expected if farmer owns their cultivated land, the variable cultivable land owned was however insignificant in all switching regression estimations. On average, farmers belonged to similar land class groups (small farms) and hence TE estimates were unable to capture the efficiency gained due to optimal operation size of tillage. Household size and off-farm income also had statistically insignificant influences on CT adoption and the TE scores of CT and TT farmers. A delay in the wheat sowing date was found to have a negative and significant effect on efficiency, exemplified by the fact that a 2–4 day delay in sowing was found to negatively affect TE by approximately 10% points. Our data therefore provide backing for studies supporting the potential of CT to accelerate sowing by reducing time-consuming tillage and crop establishment operations, with potential knock-on positive effects on yield and efficiency (cf. Keil et al. 2015; Krupnik et al. 2015a, 2015b). The effect of early sowing achieved with zero-tillage on the TE of wheat farmers in the western IGP was also reported by Krishna and Veettil (2014). However, delays in sowing may can also be experienced be due to the relative scarcity of CT machinery, which was widely reported by surveyed farmers in the study area. This can inadvertently limit the potential efficiency gains offered by CT. On the other hand, ownership of draught animals had a negatively and significant impact (*P* ≤ 0.05) on the TE of CT. With each additional number of draught animals owned, the probability of CT adoption reduces by 3.2%. This could be due to the fact that draught animals, which are mainly used for primary tillage, become comparatively less economically relevant if wheat sowing is performed by alternative machinery. Crop residues are also commonly removed to feed draught animals throughout the year, further reducing the incentive for ST adoption that relies on the maintenance of a mulch of crop residues (Gathala et al. 2015).

#### 5.3.1 Counterfactual effect of CT adoption on technical efficiency

Switching regression allows the use of estimated coefficients for the CT adopters (outcome Eq. 2) to predict the meta-frontier TE values for CT non-adopters, if the latter were to adopt the CT, and vice versa. Table 7 presents the predicted values from the regression, and the estimated counterfactual average meta-frontier TE. This table also compares the predicted values from the regression with the actual TE scores obtained from the DEA meta-frontier estimation. Further, the counterfactual scenarios for CT and TT are visually displayed using density plots (Figs. 4 and 5). These results indicate that the average predicted meta-frontier technical efficiency for TT farmers in their hypothetical regime (0.80) is significantly higher (*P* =0.00) than their observed TE. CT adopters would likely lose TE (−10%) if they *dis*adopt the technology, a point reinforced by the estimate that the counterfactual TE of CT adopters is 13% lower than their estimated DEA efficiency. Conversely, counterfactual TE of non-adopters is 23% greater than their DEA efficiency. In monetary terms, the additional profit due to efficiency gain had TT farmers adopted CT was estimated to be 14 USD ha^−1^ or 21 USD ha^−1^ had the adopters conversely *not* adopted CT. Importantly, without the effect of CT, the counterfactual TE of CT adopters is 1.54% higher than the estimated DEA efficiency of TT farmers, suggesting that adopters may be better wheat farmers, *ceteris paribus*. This finding is consistent with the general technology adoption literature indicating that initial adopters may have better farming abilities (Sunding and Zilberman 2001).

**Table 7:** Counterfactual effect of CT adoption on TE and gross margin

Nature of farmers and estimation approach	(1) Mean (SD)	(2) Min	(3) Max	(4) Counterfactual economic loss/gain USD ha^−1^)
TE of adopters if not adopted^a^	0.66 (0.05)	0.43	0.75	−21 USD ha^−1^ (−13%)
TE of adopters predicted^b^	0.76 (0.09)	0.50	0.94	No change
TE of adopters from DEA estimation^c^	0.76 (0.12)	0.46	0.95	No change
TE of non-adopters if adopted^a^	0.80 (0.05)	0.72	0.89	+13.8 USD ha^−1^ (+23%)
TE of non-adopters predicted^b^	0.65 (0.05)	0.57	0.74	No change
TE of non-adopters from DEA estimation^c^	0.65 (0.05)	0.57	0.74	No change

*Notes*: In column 1, values in parenthesis show standard deviations of mean, while those in column 4 shows % increase or decrease in gross margin

a Hypothetical counterfactual situation

b Predicted from switching regression outcome equation

c Estimated from the input-output DEA model

Finally, we also examined the impact of individual tillage options on the profitability of wheat farmers. The average gross margin for wheat production under CT adoption and TT was estimated to be approximately USD 161 ha^−1^ and USD 60 ha^−1^, respectively (Table 8), indicating important production cost advantages as also observed by Krishna and Veettil (2014) and Keil et al. (2015). The former value is an average of the estimated gross margin from the three CT options studied. When segregated, adoption of CT could achieve additional profits from wheat farming by USD 114, 127 and 75 ha^−1^, respectively for BP, PTOS and ST. By removing the inefficiencies in production, farmers can potentially operate at the frontier with projected gross margins of USD 229, 243,182 and 92 ha^−1^, respectively for BP, PTOS, ST and TT farmers. The corresponding projected gross margin for CT with efficiency improvement would be USD 212 ha^−1^. These results reveal the high potential impact of CT on the profitability of wheat farmers in the eastern IGP, and are evidenced by a 91% in commercial sales of the PTOS in Bangladesh since 2014 (CSISA-MI 2016). The corresponding benefit cost ratios (BCR) for BP, PTOS and ST are 1.81, 1.95, and 1.64 respectively; compared to 1.20 for TT. As one would expect from the TE scores, farmers who continue cultivating wheat under TT, instead of adopting one of the CT practices, will tend to underperform in terms of their BCR, with important implications for poverty reduction.

**Table 8:** Efficiency and productivity impacts of tillage options on wheat farm income

Tillage	Technical efficiency (Mean meta-frontier)	Agronomic productivity (t ha−1)	Gross margin (USD ha−1)^a^	Benefit cost ratio (BCR)
BP	0.76	4.11	174.00	1.81
PTOS	0.77	4.14	187.00	1.95
ST	0.74	3.79	135.00	1.64
CT	0.76	4.01	161.00	1.78
TT	0.65	3.68	60.00	1.20

a 1 US$ = 81.66 BDT as per the exchange rates during the time of the survey (April 2012)

BP Bed planting, *PTOS* Power tiller operated seeding, *ST* Strip tillage, *TT* Traditional tillage, *CT* Conservation tillage

## 6 Study limitations

Though CT technologies are currently being promoted in many developing countries, it should be noted that the results of this study are likely most applicable to smallholder rice-wheat systems in the eastern IGP. This is due to the comparable agro-climate and similarities with regard to agricultural practices, demographics, and other socio-economic factors. In addition, since the application of CT by Bangladeshi farmers is still in its infancy, our sample size was relatively small, which may to some extent decrease the precision of the estimation of various parameters, although controlling for endogeneity, potential selection bias, and bias in efficiency estimates partially mitigated this effect. Another limitation was the single-season nature of our study, and that labor market imperfections and various constraining biophysical factors in wheat production that exist in the eastern IGP were not explicitly introduced in the TE model. As such, future research that investigates these factors with panel data and in conjunction with a larger sample as more farmers adopt CT is desirable.

## 7 Conclusions

Viewed as an attractive option to break the current stagnation in productivity increases for the major cereal crops, while also addressing the negative environmental consequences of agriculture, interest in conservation tillage is growing globally and in South Asia. Using farmer survey data from three districts in northwest Bangladesh, we evaluated the impact of adopting three CT and machine-aided crop establishment options compared to traditional tillage with seed broadcasting by hand, in terms of their technical efficiency. Our results clearly indicate positive impacts of CT adoption on wheat farmers’ level of technical efficiency. Based on the average meta-frontier technical efficiency scores of CT adopters (0.76) and non-adopters (0.65), input use could be reduced by 24 and 35%, respectively, while maintaining the current wheat output, given that farmers are able to access the respective CT machineries. This suggests that significant gains in technical efficiency can be realized by TT farmers by switching to CT practices. Of the four tillage types studied (including TT), the PTOS was observed to be the most technically efficient option, with an average meta-technology ratio of 0.90. This was closely followed by BP (0.88) and ST (0.83), with TT lagging well behind (0.67). The results indicate that the shift from TT to PTOS may be the best option for wheat farmers from the technical efficiency perspective, though further research is needed to evaluate these findings in consideration of potential environmental trade-offs and with respect to yield and TE stability over multiple seasons.

A major advantage of CT appears to be the facilitation of early wheat sowing, which can assist farmers in escaping from terminal heat stress that adversely impacts yield and hence efficiency. Delays in sowing among CT wheat farmers is still nonetheless common, as machinery is only recently becoming commercially available at a scale that can propel rapid increases in adoption. Our results also indicate importance of farmers’ proximity and access to extension advice, and of adequate training on CT practices to improve technical efficiency. As expected, farmers *dis*adopting CT are also likely to lose technical efficiency by 10% given *their* counterfactual scenario. It is also interesting to observe that without the effect of CT, the counterfactual TE of CT adopters is 1.5% higher than the estimated DEA efficiency of the TT farmers, suggesting that adopters may be better wheat farmers, *ceteris paribus*.

Perhaps most importantly from the perspective of farmers, CT adoption is potentially more profitable (gross margin USD 161 ha^−1^, on average) than TT (mean of 60 ha^−1^). Thus, farmers adopting CT could realize substantial increases in profits: on average, a 190% increase in gross margin ($174 ha^−1^) was estimated by switching to BP, with 212% (USD187 ha^−1^) and 125% (USD135 ha^−1^) by switching to PTOS and ST, respectively. These results provide support to on-going research and development initiatives that encourage farmers to experiment with CT options in the eastern Indo-Gangetic Plains, since the three CT options analyzed in our study appear to offer large opportunities for agricultural productivity growth among smallholder farmers.

## Footnotes

1 The farmers practicing traditional tillage (TT) and ‘CT non-adopters’ are one and the same.

2 The “rDEA package” in R (version 3.02) was used for the analysis of group and meta-frontiers.

3 The threshold TE score refers to the mean TE score of the entire sample without technology differentiation; it is automatically generated in bean density plots, and is represented by a dotted line in Fig. 3B.

4 Simar and Zelenyuk (2006) demonstrated that the non-parametric test of equality of the distribution of DEA TE between two groups is more robust than testing means alone.

## References

[cit0001] Abdalla M, Osborne B, Lanigan G, Forristal D, Williams M, Smith P, Jones MB (2013) Conservation tillage systems: a review of its consequences for greenhouse gas emissions. Soil Use Manag 29 (2):199–209

[cit0002] Alene A, Manyong VM (2007) The effect of education on agricultural productivity under traditional and improved technology in northern Nigeria: An endogenous switching regression analysis. Emp Econ 32:141–159

[cit0003] Aravindakshan S, Rossi FJ, Krupnik TJ (2015) What does bench-marking of wheat farmers practicing conservation tillage in the eastern Indo-Gangetic Plains tell us about energy use efficiency? An application of slack-based data envelopment analysis. Energy 90:483–493

[cit0004] Banker RD, Charnes A, Cooper WW (1984) Some models for estimating technical and scale inefficiencies in Data Envelopment Analysis. Manag Sci 30(9):1078–1092

[cit0005] Battese GE, Rao DSP, O’Donnell CJ (2004) A metafrontier production function for estimation of technical efficiencies and technology gaps for firms operating under different technologies. J Prod Anal 21(1):91–103

[cit0006] BBS (2014) Yearbook of agricultural statistics-2012. Bangladesh Bureau of Statistics. Dhaka ISBN-978-984519-058-9

[cit0007] Bravo-Ureta BE, Greene W, Solís D (2012) Technical efficiency analysis correcting for biases from observed and unobserved variables: an application to a natural resource management project. Emp Econ 43(2012):55–72

[cit0008] Cook WD, Tone K, Zhu J (2014) Data envelopment analysis: choosing a model. Omega 44:1–4

[cit0009] CSISA-MI (2016) The Cereal Systems Initiative for South Asia – Mechanization and Irrigation (CSISA-MI) Annual Report (Oct 15 to Oct 16). International maize and Wheat Improvement Center. CIMMYT, Dhaka, Bangladesh

[cit0010] Derpsch R, Friedrich T, Kassam A, Hongwen L (2010) Current status of adoption of no-till farming in the world and some of its main benefits. Int J Agric Bio Eng 3(1):1–25

[cit0011] Erenstein O (2009) Zero tillage in the rice-wheat systems of the Indo-Gangetic plains. IFPRI Discussion Paper 00916, November 2009. http://conservationagriculture.org/uploads/pdf/ZT_WHEAT_STUDY_INDIA_IFPRI_-_ERENSTEIN_2009.pdf. Accessed 14 Sept 2014

[cit0012] Erenstein O, Thorpe W (2011) Livelihoods and agro-ecological gradients: a meso-level analysis in the Indo-Gangetic Plains, India. Agric Syst 104(1):42–53

[cit0013] FAO (2014) The State of Food and Agriculture 2014: Innovation in family farming. FAO, Rome http://www.fao.org/3/a-i4040e.pdf. Accessed 18 June 2016

[cit0014] Färe RS, Grosskopf S, Lovell CAK (1985) The measurement of efficiency of production, 1st ed. Kluwer-Nijhoff Publishing , Boston

[cit0015] Färe RS, Grosskopf S, Lovell CAK (1994) Production frontiers. Cambridge University Press, Cambridge

[cit0016] Farrell MJ (1957) The measurement of productive efficiency. J R Stat Soc Ser A (General) 120(3):253–290

[cit0017] Gathala MK, Timsina J, Islam MS, Krupnik TJ, Bose TK, Islam N, Rahman MM, Hossain MI, Harun-Ar-Rashid M, Ghosh AK, Khayer A, Tiwari TP, McDonald A (2016) Productivity, profitability, and energy: multi-criteria assessments of tillage and crop establishment options for maize in Bangladesh. Field Crops Res 186:32–46

[cit0018] Gathala MK, Timsina J, Islam S, Rahman M, Hossain I, Harun-Ar-Rashid, Krupnik TJ, Tiwari TP, McDonald AJ (2015) Conservation agriculture based tillage options can increase farmers’ profits in South Asia’s rice-maize systems: evidence from Bangladesh. Field Crops Res 172:85–98

[cit0019] Gkypali A, Tsekouras K (2015) Productive performance based on R&D activities of low-tech firms: an antecedent of the decision to export? Econ Innov New Tech 24(8):801–828

[cit0020] González-Flores M, Bravo-Ureta BE, Solís D, Winters P (2014) The impact of high value markets on smallholder productivity in the Ecuadorean Sierra: a stochastic production frontier approach correcting for selectivity bias. Food Policy 44:237–247

[cit0021] Greene W (2010) A stochastic frontier model with correction for sample selection. J Prod Anal 34:15–24

[cit0022] Hausman JA (1978) Specification tests in econometrics. Econometrica 46:1251–1272

[cit0023] Hayami Y, Ruttan VW (1985) Agricultural development: An international perspective. Johns Hopkins University Press, Baltimore, MD

[cit0024] Heckman J (1979) Sample selection as a specification error. Econometrica 47:153–161

[cit0025] Heimlich RE (1985) Land ownership and the adoption of minimum tillage: comment. Ame J Agric Econ 67:679–681

[cit0026] Keil A, D’souza A, McDonald A (2015) Zero-tillage as a pathway for sustainable wheat intensification in the Eastern Indo-Gangetic Plains: does it work in farmers’ fields? Food Sect 7:983–1001

[cit0027] King G, Nielsen R (2016) Why propensity scores should not be used for matching. Working paper. http://gking.harvard.edu/files/gking/files/psnot.pdf?m=1452103696. Accessed 4 June 2016

[cit0028] Kneip A, Park B, Simar L (1998) A note on the convergence of nonparametric DEA efficiency measures. Econom Theor 14:783–793

[cit0029] Krishna V, Aravindakshan S, Chowdhury A, Rudra B (2012) Farmer access and differential impacts of zero tillage technology in the subsistence wheat farming systems of West Bengal, India. CIMMYT Socio-Economics Working Paper 7. CIMMYT, Mexico, D.F

[cit0030] Krishna V, Keil A, Aravindakshan S, Meena M (2017) Conservation tillage for sustainable wheat intensification: the example of South Asia. In Achieving sustainable cultivation of wheat, Vol. 2, Burleigh Dodds Science Publishing Limited, pp 1–22

[cit0031] Krishna VV, Veettil PC (2014) Productivity and efficiency impacts of conservation tillage in northwest Indo-Gangetic Plains. Agric Syst 127:126–138

[cit0032] Krupnik TJ, Ahmed ZU, Timsina J, Shahjahan M, Kurishi ASMA, Rahman S, Miah AA, Gathala MK, McDonald AJ (2015a) For-going the fallow in Bangladesh’s stress-prone coastal deltaic environments: effect of sowing date, nitrogen, and genotype on wheat yield in farmers’ fields. Field Crops Res 170:1–7

[cit0033] Krupnik TJ, Ahmed ZU, Timsina J, Yasmin S, Hossain F, Mamun A, McDonald AJ (2015b) Untangling crop management and environmental influences on wheat yield variability in Bangladesh: an application non-parametric approaches. Ag Syst 139:166–179

[cit0034] Krupnik TJ, Santos Valle S, Hossain I, Gathala MK, Justice S, Gathala MK, McDonald AJ (2013) Made in Bangladesh: Scale-appropriate machinery for agricultural resource conservation. International Maize and Wheat Improvement Center, Mexico, D. F, p 126

[cit0035] Li Q, Maasoumi E, Racine JS (2009) A nonparametric test for equality of distributions with mixed categorical and continuous data Journal of Econometrics 148(2):186–200

[cit0036] Lin M, Huybers MP (2012) Reckoning wheat yield trends. Env Res Lett 7(024016):1–6

[cit0037] Lokshin M, Sajaia Z (2004) Maximum likelihood estimation of endogenous switching regression models. Stata J 4(3):282–289

[cit0038] Lovell CAK (1993) Production frontiers and productive efficiency. In: Fried HO Schmidt SS and Lovell CAK (eds) The Measurement of productive efficiency: techniques and applications, Oxford University Press, Oxford UK, pp 3–67

[cit0039] Maddala GS (1983) Limited dependent and qualitative variables in econometrics. Cambridge University Press, Cambridge

[cit0040] Maddala GS, Nelson FD (1975) Switching regression models with endogenous and exogenous switching. American Statistical Association Proceedings of the Business and Economic Statistics Section, pp 423–426

[cit0041] Mitchell JP, Pettygrove GS, Upadhyaya S, Shrestha A, Fry R, Roy R, Hogan P, Vargas R, Hembree K (2009) Classification of conservation tillage practices in California irrigated row crop systems. Pub. 8364. University of California, Division of Agricultural and Natural Resources, Oakland, California, USA

[cit0042] Mottaleb KA, Krupnik TJ, Erenstein O (2016) Factors associated with small-scale agricultural machinery ownership in Bangladesh: census findings. J Rural Stud 46:155–168 27524857 10.1016/j.jrurstud.2016.06.012 PMC4973803

[cit0043] O’Donnell CJ, Rao DP, Battese GE (2008) Metafrontier frameworks for the study of firm-level efficiencies and technology ratios. Empi Econ 34(2):231–255

[cit0044] Olesen OB, Petersen NC (1995) Chance constrained efficiency evaluation. Manag Sci 41:442–457

[cit0045] Perloff JM, Lynch L, Gabbard D (1998) Migration of seasonal agricultural workers. Am J Agric Econ 80:154–164

[cit0046] Qureshi A, Ahmed ZU, Krupnik TJ (2015) Moving from resource development to resource management: problems, prospects and policy recommendations for sustainable groundwater management in Bangladesh. Water Res Mgt 29:4269–4283

[cit0047] Raghu PT, Aravindakshan S, Rossi F, Krishna V, Baksh E, Miah AA (2016) A biophysical and socioeconomic characterization of the cereal production systems of northwest Bangladesh. Cereal Systems Initiative for South Asia project, Phase III. CIMMYT, Dhaka, Bangladesh

[cit0048] Rahman S, Wiboonpongse A, Sriboonchitta S, Chaovanapoonphol Y (2009) Production efficiency of Jasmine rice producers in northern and north-eastern Thailand. J Agric Econ 60:419–435

[cit0049] Ramalho EA, Ramalho JJS, Henriques PD (2010) Fractional regression models for second stage DEA efficiency analyses. J Prod Anal 34:239–255

[cit0050] Rehman H, Nawaz A, Wakeel A, Saharawat Y, Farooq M (2014) Conservation agriculture in South Asia. Farooq M, Siddique KHM (eds) Conservation Agriculture. Springer International Publishing, Switzerland, pp 249–283. 10.1007/978-3-319-11620-4_11

[cit0051] Ruttan VW, Hayami Y (1984) Toward a theory of induced institutional innovation. J Dev Stud 20(4):203–223

[cit0052] Seiford LM, Thrall RM (1990) Recent developments in DEA: the mathematical programming approach to frontier analysis. J Econ 46(1):7–38

[cit0053] Simar L (1992) Estimating efficiencies from frontier models with panel data: a comparison of parametric, non-parametric and semiparametric methods with bootstrapping. J Prod Anal 3:167–203

[cit0054] Simar L, Wilson PW (2000) A general methodology for bootstrapping in non-parametric frontier models. J Appl Stat 27(6):779–802

[cit0055] Simar L, Wilson PW (2007) Estimation and inference in two-stage, semi-parametric models of production processes. J Econ 136 (1):31–64

[cit0056] Simar L, Wilson PW (2011) Two-stage DEA: caveat emptor. J Prod Anal 36(2):205–218

[cit0057] Simar L, Zelenyuk V (2006) On testing equality of distributions of technical efficiency scores. Econ Rev 25(4):497–522

[cit0058] Solís D, Bravo-Ureta BE, Quiroga R (2007) Soil conservation and technical efficiency among hillside farmers in Central America: a switching regression model. Aust J Agric Resour Econ 51:491–510

[cit0059] Sunding D, Zilberman D (2001) The agricultural innovation process: research and technology adoption in a changing agricultural sector. In: Gardner BL, Rausser GC (eds) Agricultural production, handbook of agricultural economics, vol 1 Elsevier, New York, pp 207–261

[cit0060] Villano R, Bravo-Ureta BE, Solís D, Fleming E (2015) Modern rice technologies and productivity in the Philippines: disentangling technology from managerial gaps. J Agric Econ 66:129–154

[cit0061] Wadud A (2003) Technical, allocative and economic efficiency of farms in Bangladesh: a stochastic frontier and DEA approach. J Dev Areas 37:109–126

[cit0062] Wollni M, Brümmer B (2012) Productive efficiency of specialty and conventional coffee farmers in Costa Rica: accounting for technological heterogeneity and self-selection. Food Policy 37:67–76

[cit0063] World Bank (2016) Population density (people per sq. km of land area). http://data.worldbank.org/indicator/EN.POP.DNST Accessed 16 July 2016

