# Supplementary figures and images for: Application of a bias-corrected meta-frontier approach and an endogenous switching regression to analyze the technical efficiency of conservation tillage for wheat in South Asia
Source: J Product Anal. 2018 Feb 5;49:153–71. doi: 10.1007/s11123-018-0525-y (PMC7771628; doi:10.1007/s11123-018-0525-y)

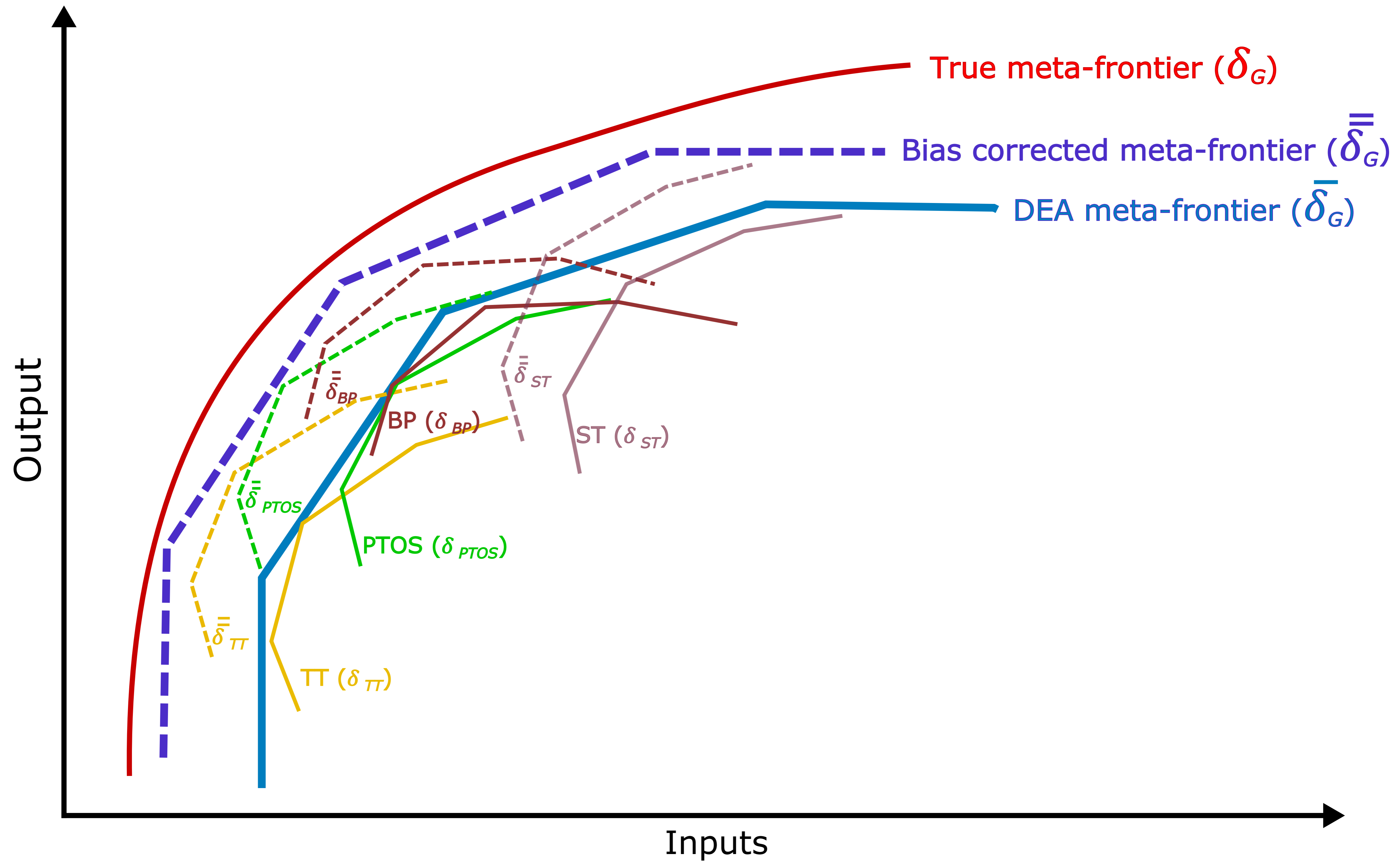

Supplement: Supplementary file 1 [file JPA-49-153-s001.pdf]
