# Supplementary material for: Application of a bias-corrected meta-frontier approach and an endogenous switching regression to analyze the technical efficiency of conservation tillage for wheat in South Asia
Source: J Product Anal. 2018 Feb 5;49:153–71. doi: 10.1007/s11123-018-0525-y (PMC7771628; doi:10.1007/s11123-018-0525-y)
Supplement: Supplementary file 2 [file JPA-49-153-s002.docx]

Supplementary Material – S1

The bias corrected DEA meta-frontier is graphically represented in Figure S1. Group specific frontiers for each group *k*, represents the $\delta_{k}$ where $\delta_{k}=\left\{ \delta_{TT},\delta_{PTOS},\delta_{BP},\delta_{ST} \right\}$ There are three meta-frontier curves in the graphs: first curve represents the true meta-frontier, $\delta_{G}$; the second frontier represents the DEA meta frontier, $\bar{\delta}_{G}$ and the third frontier represents the bias corrected DEA metafrontier, $\delta_{G}$. The distance between true meta-frontier and DEA meta-frontier is the total bias ($b_{total})$. Using a bootstrap approach (Simar and Wilson, 2007) described in section 3.1.1., the bias is estimated for each group specific frontier *k* ($\hat{b}_{k})$ as well as for DEA metafrontier ($\hat{b}_{G})$. The dotted production frontiers correspond to the bias corrected alternatives of tillage specific ($\delta_{k}=\left\{ \delta_{TT},\delta_{PTOS},\delta_{BP},\delta_{ST} \right\})$as well as DEA meta-frontiers${(\delta}_{G})$.
